# Power of mentorship for civilian and military acute care surgeons: identifying and leveraging opportunities for longitudinal professional development

**DOI:** 10.1136/tsaco-2022-001049

**Published:** 2023-02-27

**Authors:** Lisa Marie Knowlton, William Jason Butler, Ryan Peter Dumas, Brittany K Bankhead, Jonathan P Meizoso, Brandon Bruns, Jan-Michael Van Gent, Haytham M A Kaafarani, Matthew J Martin, Nicholas Namias, Deborah M. Stein, Matthew D Tadlock, R Shayn Martin, Kristan L Staudenmayer, Jennifer M Gurney

**Affiliations:** 1 Division of General Surgery, Section of Acute Care Surgery, Stanford University, Stanford, California, USA; 2 Stanford University School of Medicine, Department of Surgery, Stanford, California, USA; 3 General Surgery, US Naval Hospital Camp Pendleton, Oceanside, California, USA; 4 Surgery, UT Southwestern Medical, Dallas, Texas, USA; 5 Division of Trauma, Burns, and Critical Care, Texas Tech University Health Sciences Center, Lubbock, Texas, USA; 6 DeWitt Daughtry Family Department of Surgery, Ryder Trauma Center, Jackson Memorial Hospital, University of Miami Miller School of Medicine, Miami, Florida, USA; 7 Department of Surgery, UT Southwestern Medical School, Dallas, Texas, USA; 8 Division of Trauma and Surgical Critical Care, The University of Texas Health Science Center at Houston, Houston, Texas, USA; 9 Department of Surgery, Massachusetts General Hospital, Boston, Massachusetts, USA; 10 Division of Trauma and Surgical Critical Care, LAC USC Medical Center, Los Angeles, California, USA; 11 Department of Surgery, Program in Trauma, R Adams Cowley Shock Trauma Center, University of Maryland School of Medicine, Baltimore, Maryland, USA; 12 1st Medical Battalion, 1st Marine Logistics Group, US Naval Hospital Camp Pendleton, Camp Pendleton, California, USA; 13 Department of Surgery, Division of Acute Care Surgery, Wake Forest School of Medicine, Winston-Salem, North Carolina, USA; 14 Division of General Surgery, Section of Acute Care Surgery, Stanford University School of Medicine, Stanford, California, USA; 15 Department of Trauma Surgery, San Antonio Military Medical Center, Fort Sam Houston, Texas, USA

**Keywords:** education, medical, general surgery, teaching

## Abstract

Across disciplines, mentorship has been recognized as a key to success. Acute care surgeons, focused on the care of trauma surgery, emergency general surgery and surgical critical care, practice in a wide variety of settings and have unique mentorship needs across all phases of their career. Recognizing the need for robust mentorship and professional development, the American Association for the Surgery of Trauma (AAST) convened an expert panel entitled ‘The Power of Mentorship’ at the 81st annual meeting in September 2022 (Chicago, Illinois). This was a collaboration between the AAST Associate Member Council (consisting of surgical resident, fellow and junior faculty members), the AAST Military Liaison Committee, and the AAST Healthcare Economics Committee. Led by two moderators, the panel consisted of five real-life mentor-mentee pairs. They addressed the following realms of mentorship: clinical, research, executive leadership and career development, mentorship through professional societies, and mentorship for military-trained surgeons. Recommendations, as well as pearls and pitfalls, are summarized below.

## Introduction

The art of ‘mentor acquisition’in any field remains an elusive concept, with significant variability in the adoption of structured versus organic relationships that develop more naturally throughout an individual’s career, and an occasional challenge in pragmatic implementation.^1 2^ The path to becoming an acute care surgeon, focused on the care of trauma surgery, emergency general surgery and surgical critical care patients, is an arduous one involving multiple years of formal education followed by residency training and fellowship and can be complicated by many different practice models. Mentorship does not end at the completion of training, rather, it could be argued that it is more critical in the early stages of practice; longitudinal mentorship and professional development is necessary for a productive, rewarding, and successful career. Successful surgical mentors are ones that deliberately make the time to listen, hold regular (and impromptu) meetings, promote mentees, support them during failures, and celebrate their successes.^3 4^ A successful mentee develops clear career goals, sets expectations, helps drive the relationship, and delivers on their end of the relationship by being productive.^5^ However, mentorship for acute care surgeons is not ‘one-size-fits-all’. Practices vary significantly regarding patient mix, clinical setting, academic, administrative or leadership pursuits and for surgeons who are military trained.

**Figure 1:**
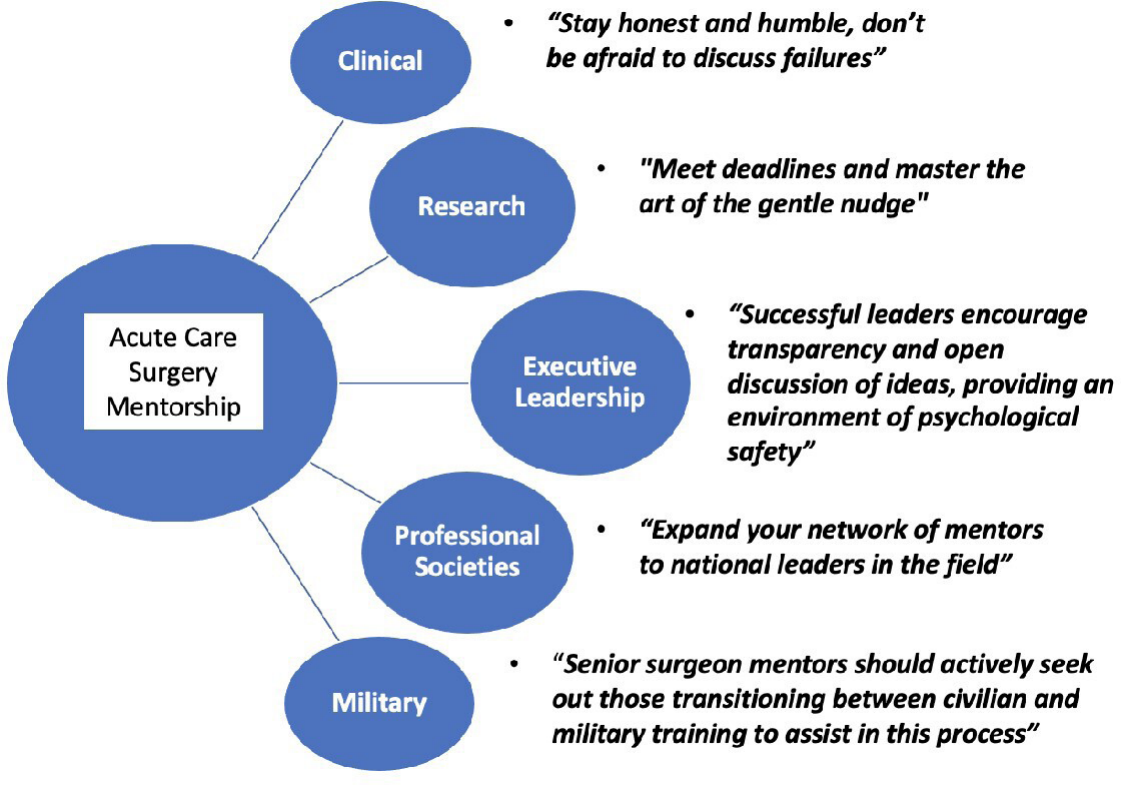
Quotes from the 2022 81st AAST annual meeting acute care surgery mentorship panel.

Recognizing the need for robust, diverse mentorship and professional development as acute care surgeons, the national professional organization for acute care surgeons—the American Association for the Surgery of Trauma (AAST)—convened an expert panel entitled ‘The Power of Mentorship’ at the 81st annual meeting in September 2022 (Chicago, Illinois). This was a collaboration between the AAST Associate Member Council (consisting of surgical resident, fellow and junior faculty members of the organization), the AAST Military Liaison Committee and the AAST Healthcare Economics Committee. Led by two moderators, the panel consisted of experienced and ‘models of success’ mentor-mentee pairs who engaged in an interactive discussion (with each other and the audience) of the most important considerations of mentorship. Select significant quotes from the panel are represented in figure 1. The aim was to address the following realms of mentorship: clinical, research, executive leadership and career development, mentorship through professional societies and mentorship for military-trained surgeons. Recommendations, as well as pearls and pitfalls, are provided in each of these areas.

## Clinical mentorship

Surgical trainees often assume that the end of training signals full independence and that they have lost the privilege of clinical advice and help from their educators and mentors when, in reality, mentorship is crucial for young faculty as they launch their careers. Trusted mentors are imperative to providing ongoing guidance regarding challenging cases, intraoperative consultation as needed as well as providing a safe space for surgeons to discuss successes, failures, and potential barriers to establishing independent and productive career paths. When identifying a clinical mentor, two steps are crucial.

First, the mentee must seek out a surgeon whose clinical practice they respect and perhaps even want to emulate. The mentor and mentee do not necessarily need to have the same clinical specialty, but the mentor must be someone whose clinical judgment the mentee trusts and admires. If clinical mentoring partnerships occur across institutions, agreeing a priori on a mode of communication (eg, text messages, phone calls, emails, video chat, zoom meetings) streamlines the relationship.^6^ Being receptive for evolution of modes of communication as careers evolve is also helpful and allows the relationship to grow and adapt to the mentees needs

Second, the mentor and mentee must both commit to having open and honest communication. The eagerness to impress one’s mentor can subconsciously lead to concealing or minimizing of struggles, avoiding discussing poor outcomes, or hiding one’s anxiety regarding difficult cases. Discussion of both clinical successes, failures, and transparent opportunities for improvement is of paramount importance. The onus of sharing these transparently is on the mentee. While sharing accolades and achievements may be against surgical culture and the nature of most junior faculty, they should do so to allow the mentor to celebrate these successes with them and guide them towards potential future endeavors.

Similarly, a mentor should be willing to share their own prior clinical challenges and how they learned from them. This creates an environment in which mentees can feel safe to disclose their own perceived failures or shortcomings and leads to better self-reflection regarding opportunities for technical improvement along with better recognition of opportunities for filling career gaps. Overall, this helps the mentee enter into a growth mindset. Further, the combination of a self-aware mentee with an empathetic and thoughtful mentor will lead to a healthy, mutually beneficial relationship that improves patient care via the provision of sound clinical advice and the improved performance of the mentee.

## Research mentorship

Selecting a research mentor requires introspection by the prospective mentee. Mentees need to consider the reasons for conducting research, the type of research they will conduct (basic science, translational, etc), presence of competing clinical or educational responsibilities, and perhaps most important, which subject matter truly interests them. These considerations can help mentees to identify potential mentors at their institution or mentors located remotely. Online faculty profiles can be used to identify faculty with similar interests, evaluate their academic productivity, and assess how frequently they support mentees. This should be followed by an in-person or video meeting to explore and gauge the interest and ability of a prospective mentor to commit the time necessary for a successful mentor/mentee relationship.

Mentees must be responsible for their own academic productivity for the relationship to succeed. As the mentee, commitment of time and effort required to produce high-quality research is needed.^7^ Mentees must establish a system of communication that works best for them and their mentors, whether through regular meetings or electronic communication. Mentees should be respectful of mentors’ time by coming to meetings well prepared and by meeting agreed on deadlines. A summary of pearls and pitfalls are summarized in table 1. The mentor should be invigorated by the mentee’s level of commitment and proactivity rather than experiencing an undue burden of progressing them forward. Additionally, mentors are often busy, so mentees, will need to learn the art of the *gentle nudge*, that is, sending mentors a tactful reminder that something is sitting in their inbox and a deadline is approaching. Finally, remember that the goal of the relationship is professional development. Mentees must be open to and seek feedback; using this valuable time to learn and hone their craft as surgeon-scientists.

**Table 1:** Pearls and pitfalls across mentorship categories

Mentorship categories	Pearls	Pitfalls
Clinical	Select a mentor who is relatable in terms of clinical practice. Cultivate a psychologically safe environment to discuss challenging case or request intraoperative guidance.	Failure of the mentor or mentee to openly and honestly acknowledge successes and failures. Self-critical or punitive attitudes toward clinical complications.
Research	Maximize meeting productivity with a focused agenda. Keep the mentee’s best interest in mind in terms of authorship, recognition, promotion.	Engaging in a research mentorship when one or both parties lack bandwidth. Failure for both parties to set clear goals and meet deadlines.
Executive leadership	Identify mentors who have followed a similar leadership pathway (eg, advanced degree, administrative role, etc) to understand the nature of the commitment. Mentors should help seek out leadership and job opportunities, committee participation, and national or global engagement.	Not being open to ongoing mentorship as one progresses through their career or inability to consider ‘non-traditional’ opportunities. Choosing leadership roles, pathways, or mentors that do not align with one’s interests, strengths, and academic mission.
Professional societies	Be strategic and pragmatic in selecting the organization that best fits your needs and try to diversify your mentors across institutions. Identify at least one ‘hard target’ or deliverable from committee or professional society engagement.	Avoid overcommitting to multiple mentors or committees and not being able to deliver. Do not use professional society mentorship as a substitute for local mentorship, as the mentor will lack specific insight for local programmatic issues.
Military	Find an experienced surgeon who can help navigate a long-term career, transitions between civilian and military practice, and leverage opportunities across professional surgical societies where indicated. Whether planning a long-term or short-term career, seek out deployment or overseas experiences as these can be challenging and incredibly rewarding.	Failure to obtain a clear sense of the metrics for success and promotion in your current military or civilian setting. Failure to maintain clinical proficiency or seek opportunities when off duty or not deployed.

For mentors, the most important initial considerations are whether they have the time and skill set to devote to prospective research mentees. Without a careful evaluation of these two factors, the relationship may be destined for failure from the start. Mentors should also be clear regarding expectations. Each meeting should generate a list of deliverables that will provide structure and guidance. Establishing open lines of communication, thoughtfully reviewing work, and providing quality feedback are important components of the research partnership. Finally, and most importantly, place the best interests of mentees first. If they did the work, let them be the first author, and consider their career goals when choosing a journal or meeting. There is a difference between a research assistant and a mentee. Let mentees shine—the successes are shared, but let the mentees be the ‘stars’. Mentors shine in the light that comes from them!

Some surgeons may find themselves searching for the next professional challenge, especially those who seek to change systems of care and support the practice of many. Those seeking to engage in surgical leadership should realize that this challenge may require additional professional development at the hospital or national level, and could necessitate specialized mentorship and advice regarding advanced degree programs and available administrative, leadership, and non-clinical roles.^8^

For the surgeon seeking a novel position that requires an additional degree or specialized training, it is prudent to identify an individual who can serve as a mentor and/or sponsor who has taken a similar pathway. Inquiring about the steps taken to achieve this role, will shed light on the investment of time and money required. Similarly, volunteering and actively seeking committee work at the hospital, regional, or national level with these individuals provides an opportunity to form relationships and gain personal and system knowledge.

For individuals pursuing an additional degree, making time to meet with a representatives, current or former students from the candidate program can provide more logistical clarity (financial and time commitments, full-time vs part-time, virtual vs in-person). Discussing the granular details of the program will give invaluable insight about the true expectations and effort required.

As surgical careers progress, opportunities may arise for administrative and other non-clinical roles. In considering whether to pursue these opportunities, it is imperative that individuals understand their values and how they obtain fulfillment. If treating patients is the surgeon’s source of joy, pursuing administrative roles that take time away from patient care may not be ideal. Similarly, if more time away from work for non-medical interests is desired, one should carefully weigh the pros and cons of taking on additional roles. When administrative and other leadership roles are desired, seeking out those opportunities by applying for open positions and discussing professional desires with mentors will serve to identify the surgeon as a candidate for available roles. Volunteering for committee work (both locally and nationally) can foster relationships, build knowledge, and provide practical experiences that help surgeons obtain and succeed in future roles and ultimately support local, regional, and national initiatives that have great impacts on improving patient care on a large scale.

In the case of more senior acute care surgeons, it is vitally important to remain open to mentorship from peers and more junior individuals who have achieved success in their area of interest. Successful leaders encourage transparency and open discussion of ideas, providing an environment of psychological safety. To ensure senior leaders do not become isolated monarchs of their organizations, seeking advice from a trusted group of advisors is recommended.^9^

Creating relationships with a variety of individuals throughout a surgical career truly is a cornerstone of a successful career. These mentoring relationships are vital for ongoing academic promotion as references from outside institutions are required. Additionally, fostering such relationships affords speaking opportunities, job and leadership opportunities, committee participation, and involvement at a national and global level. Most importantly, the relationships built throughout a career create the friendships that literally last for a lifetime.

## Mentorship through professional societies

Professional societies have adapted and changed strategic visions to attract younger and more diverse surgeons. As such, mentorship opportunities through these societies have evolved dramatically over the last decade. Opportunities that were once reserved for mid-career or senior surgeons are often available to junior faculty. For example, the AAST formed the AAST Associate Membership in 2019 to engage residents, fellows, and junior faculty in their organization. The American Board of Surgery is now recruiting new associate examiners to diversify the demographics of the examiner pool. Moreover, the use of social media for outreach and to disseminate educational and mentorship programs available through professional societies has increased considerably. Programs, such as the Eastern Association for the Surgery of Trauma ‘Mentoring Family’, readily connects surgeons at different stages in their careers across the USA. Many of these new initiatives to engage younger surgeons often include mentorship programs as a cornerstone. Direct sponsorship and mentorship of a trainee or junior faculty member by a more senior faculty often opens doors that may have otherwise been closed. A list of formal mentorship initiatives through professional surgical organizations are outlined in box 1.

Box 1 **List of mentorship programs through professional societies**

- AAST Associate Member Mentoring Scholarship
- AAST Trauma Surgery Acute Care Open Peer Review Mentorship Program
- EAST Executive Leadership Coaching and Mentoring Program
- EAST Peer Review Mentoring Program
- EAST Mentoring Family
- AAS-SUS Basic/Translational Research Mentoring Program
- American College of Surgeons Committee on Trauma Future Trauma Leaders Program
- AAS Aspiring Leaders Development Program

AAS, Association for Academic Surgery; AAST, American Association for the Surgery of Trauma; EAST, Eastern Association for the Surgery of Trauma; SUS, Society of University Surgeons.

Most recently, national mentorship and sponsorship across institutions has been made increasingly easy with the widespread adoption of virtual meetings. This allows for ‘close’ mentorship and collaboration with a broad network of mentors and mentees and helps to drive professional organization productivity. Together the mentor/mentee pair can identify ‘hard targets’ and ‘deliverables’ to work towards, whether individually or through broader organizational committee work. Mentorship through professional organizations provides exposure to, as well as support and guidance from an extensive network national expert leaders beyond one’s home institution.^10^ However, given the complexities of clinical work and academia, it remains important for faculty to identify local institutional mentor champions as well.

## Military mentorship

In addition to juggling the responsibilities of their civilian colleagues, military surgeons are often required to perform administrative and leadership duties early in their careers. Additionally, the requirements to deploy, master the principles of surgery in challenging and austere environment, as well as communicate effectively with non-medical leaders is imperative for success as a military surgeon. Not having control over where you live/practice can be an added stress of a military surgeon, especially if they are required to move every few years. How these competing priorities are ‘racked and stacked’ depends on the surgeon’s long-term military, surgical, and academic goals. For those seeking a long-term military career, maintaining the clinical excellence required to care for those who go in harm’s way can be especially challenging while maneuvering within a rapidly evolving military health system (MHS) characterized by changing priorities after 20 years of war, financial constraints, and declining skills sustainment opportunities at military hospitals.^11^

When junior surgeons are selecting their first postresidency billet, questions to consider include: Is a fellowship planned? Is the surgeon planning on transitioning to civilian practice, or are they planning a long-term career? No matter the goal, in most instances the following is true:

1. Seek out deployment, expeditionary, or overseas experiences. Caring for patients in diverse environments and those who serve our country can be incredibly rewarding and positive experience.
2. Do not make any final decisions or close doors of opportunity until the first tour is completed as it can often refocus a young surgeon’s (and their family’s) initial plan for a shorter or longer military career.

Once initial goals are determined, mentors can help with execution by giving advice on billet choices and available leadership position as well as helping the mentee find opportunities to help influence military medicine policy and doctrine. Without a strong senior surgeon advocate, this balancing act can feel near impossible and is primed to fail the surgeon and the military alike.

Senior surgeons should actively seek out those transitioning from civilian training to active service to assist in this process as they often do not have the institutional knowledge military trainees acquire during residency.^12^ Transitioning into active service can be an uphill battle with significant bureaucratic hurdles. In many commands junior surgeons are *senior officers*, without the requisite experience that comes with the rank; while this can be an opportunity, it can also be a pitfall and the right mentorship can help with these challenging transitions. Learning the military vernacular (and the many acronyms!), policies, objectives, and platforms can be a steep learning curve. Furthermore, learning how to operate in austere environments after training in academic centers with often unlimited resources and vast subspecialist capabilities, can be challenging.^13^ In some environments, just because one *can* do an operation does not mean one *should*. A senior surgeon must help quickly boil down the pearls and pitfalls, otherwise, the mentee may be set up for imminent failure (table 1).

In many military hospitals and operational commands, academic and research opportunities may not be readily available. However, the Joint Trauma System and the Defense Committees on Trauma, modeled after the American College of Surgeons Committee on Trauma, have numerous opportunities to participate in developing and revising clinical practice guidelines and to influence policy and doctrine throughout the MHS. Many academic surgical societies have active military committees and a military focus at their annual meetings that provide opportunities for young military surgeons to participate (table 2).

**Table 2:** List of surgical societies with military-specific committees, societies, and annual symposia

Organization	Opportunities for military surgeons
American College of Surgeons	Excelsior Surgical Society with an annual symposium at the Clinical Congress Dedicated military scientific abstract sessions
American Association for the Surgery of Trauma	Military liaison committee and starting in 2023 will host an annual military premeeting symposium Military scholarship to fund attendance to annual meeting
Eastern Association for the Surgery of Trauma	Combat casualty article database on website, Military Ad Hoc Committee Military scholarship to fund attendance to annual meeting
Society of American Gastrointestinal and Endoscopic Surgeons (SAGES)	Military Committee and Society of Military Surgeons conference during annual meeting Dedicated military scientific abstract sessions
Society of Critical Care Medicine	Military committee (Uniformed Services Committee)
Association of Academic Surgery	Military Committee Military scholarship to fund attendance to annual meeting Dedicated military scientific a abstract sessions
American Academy of Orthopedic Surgeons/American Association of Orthopedic Surgeons, Orthopedic Trauma Associations, Society of Military Orthopedic Surgeon, Orthopedic Research Society, American Orthopedic Society for Sports Medicine	Extremity War Injuries Symposium, Distinguished Visiting Scholars Program
Association of Military Surgeons of the United States	Military medical society focused on leadership skills and strategic planning

While academic promotion, research, and clinical productivity can positively impact a surgeon’s military career progression, these tend not to be the primary determinants for promotion in military rank. This can be a significant challenge for a military surgeon who must decide early in their career if they want to pursue clinical/academic excellence versus military leadership; since there is no clinical or academic path to senior military leadership, it is difficult to do both. The military surgeon is expected to carry out the priorities and objectives of their Commanding Officer (CO), which are often operational and wartime oriented. If one’s CO is a line officer who commands an aircraft carrier or an infantry brigade, meeting operationally focused expectations and those required of an academic career while maintaining clinical skills can be challenging.^14^ Senior surgeon mentorship is key for navigating these waters. Given the well-documented decreased availability of clinical experiences in most military hospitals, it is paramount that junior surgeons seek opportunities to maintain clinical currency and competency by either participating in military-civilian partnerships (MCP) or seeking other means to maintain clinical volume. This allows them to maintain deployment readiness *and* specialty-specific skillsets while ensuring their ability to save lives when deployed and optimize the recovery of injured soldiers, sailors, marines, and airmen at home.^14 15^ How military surgeons balance clinical viability, command responsibilities, academic/research interests, *and* promote is an ongoing challenge in military surgical careers senior surgeons must blaze the trail, advocate to military leadership the importance of clinical care, and create environments for surgeons to have MCP opportunities in general surgery, trauma, and subspecialty care when not available at local military hospitals. Additionally, support and encouragement for junior surgeons to pursue civilian academic promotion requires support and advocacy of senior military surgeons.

## Conclusion

The Acute Care Surgeon has diverse and complex mentorship needs. Successful careers are built on longitudinal mentor-mentee relationships that encompass multiple facets of career development. Wherever appropriate, mentors can be readily identified locally or across institutions. Leveraging connections made through professional societies can also lead to meaningful mentoring partnerships, impactful engagement, and promotion.
